# YB-1 Synthesis Is Regulated by mTOR Signaling Pathway

**DOI:** 10.1371/journal.pone.0052527

**Published:** 2012-12-20

**Authors:** Dmitry N. Lyabin, Irina A. Eliseeva, Lev P. Ovchinnikov

**Affiliations:** Institute of Protein Research, Russian Academy of Sciences, Pushchino, Moscow Region, Russian Federation; The John Curtin School of Medical Research, Australia

## Abstract

YB-1 is a eukaryotic protein with numerous intra- and extracellular functions based on its ability to interact with RNA, DNA, and many proteins. In spite of achievements in studying its functions, regulation of YB-1 synthesis in the cell remains poorly understood. In the current study Western and Northern blotting were used to determine the amounts of YB-1 and *YB-1* mRNA in rabbit organs and several cell lines. As found, in the majority of studied eukaryotic cells a considerable proportion of *YB-1* mRNA was stored in free mRNPs, i.e., was poorly translated. Also, we demonstrated that YB-1 synthesis depended on conditions that determined the rate of cell division. Specific suppression of YB-1 synthesis resulted from inhibition of the mTOR signaling pathway with inhibitor PP242, but not rapamycin. Experiments on reporter constructs showed that dependence of *YB-1* mRNA translation on activity of the mTOR signaling pathway was dictated by 5′ untranslated regions of this mRNA, irrelatively of the TOP-like sequences at the beginning of 5′ UTR.

## Introduction

YB-1 belongs to proteins with a cold shock domain and performs many functions in the cell (reviewed in [1]). It interacts with many other proteins and with DNA and RNA. It acts as a chaperon of nucleic acids [2], thereby restoring their conformational mobility lost upon a temperature decrease. By binding to nucleic acids, YB-1 regulates virtually all DNA- and mRNA-dependent events in eukaryotic cells, including replication and reparation of DNA [3]–[5], as well as transcription [1], splicing of mRNA [6], [7] and mRNA translation [8]–[11]. In other words, it performs both overall and specific regulation of gene expression at differential levels. The amount of YB-1 is especially high in cancer cells, which makes it a pronounced marker of tumors [12], [13]. YB-1 translocation from the cytoplasm to the nucleus stimulates transcription of a number of genes encoding protective proteins, including those responsible for multiple drug resistance [14]. When involved in DNA reparation in the nucleus, YB-1 contributes to cell resistance against ionizing radiation and xenobiotics [15]. Therefore, the nuclear localization of YB-1 is considered to be an early marker of multiple drug resistance of cancer cells [12], [16]. Its elevated concentration in the cytoplasm may prevent oncogenic transformation of the cell caused by activated PI3K/Akt kinase signaling pathway [17].

Besides, YB-1 can promote transition of differentiated epithelial cells into mesenchymal stem ones with an elevated migration ability allowing their dispersion elsewhere [18]. As shown by independent studies, an elevated expression of YB-1 in tumors correlates with enhanced tumor dissemination, which makes YB-1 an early marker of metastasis [19]–[21]. Also, YB-1 was shown to be able to secret from cells and to bind to Notch receptors on the cell surface, thereby stimulating cell proliferation similar to the epidermal growth factor [22], [23].

In spite of considerable achievements in studying YB-1 functions, regulation of YB-1 synthesis in the cell remains poorly understood. The reported studies are mostly devoted to regulation of *YB-1* expression at the level of transcription [24]–[28]. There are only a few papers on expression regulation at the post-transcriptional level [29]–[32], although it is quite possible.

Here, using Western and Northern blotting, we detected amounts of YB-1 and *YB-1* mRNA in rabbit organs and in some cultivated cell lines. As found, these two values frequently show no correlation with each other in rabbit organs. In cultivated cells, they are slightly variable, with the bulk of *YB-1* mRNA usually observed in free mRNPs, i.e., poorly translated. Using [^35^S]-Met pulse labeling of cell proteins followed by immunoprecipitation of YB-1, we determined the rate of YB-1 synthesis in the cell. It was shown to depend on conditions affecting the rate of cell division (cell culture density, serum starvation). Finally, we have shown that inhibition of the mTOR kinase results in specific negative regulation of both endogenous YB-1 synthesis and translation of reporter constructs containing *YB-1* mRNA 5′ UTR.

## Results

### Analysis of YB-1 and *YB-1* mRNA Amounts in Cultivated Cells and Rabbit Organs

**Figure 1:**
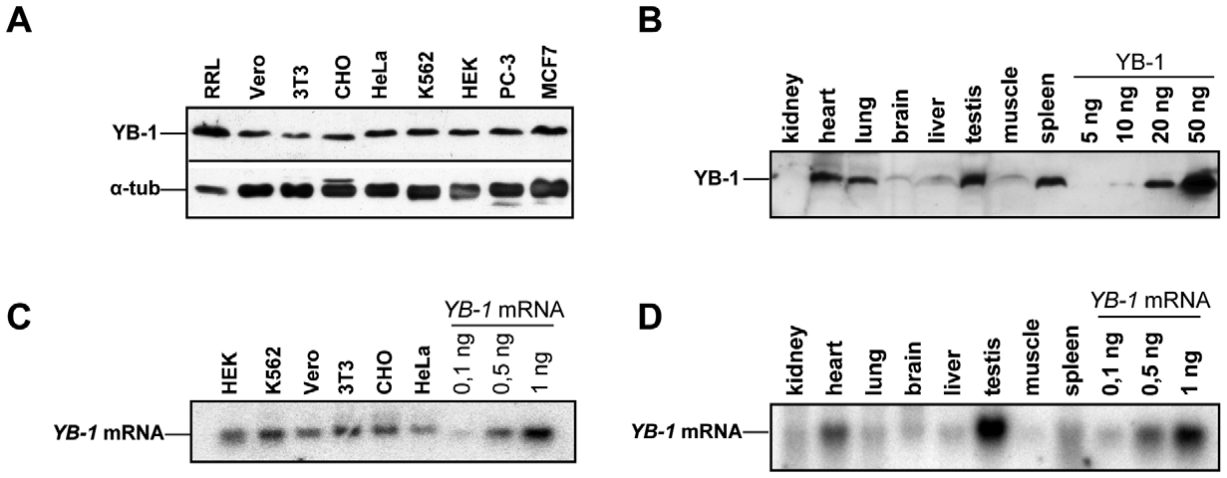
Analysis of YB-1 and *YB-1* mRNA amounts in the cell. **A** and **B,** 15 µg of total protein from lysates of various cell lines (A) or 50 µg of total protein from tissue lysates (B) were analyzed by Western-blotting. **C** and **D,** 10 µg of total RNA from lysates of various cell lines (C) or tissue lysates (D) were analyzed by Northern-blotting.

Prior to determining the level of *YB-1* mRNA translation, we estimated the amounts of YB-1 and *YB-1* mRNA in several eukaryotic cell cultures and various rabbit organs. The amount of YB-1 was determined using Western blot with antibodies against its C-terminal peptide, while Northern blot was used to detect *YB-1* mRNA. As seen from Fig. 1A, in total lysates of eukaryotic cell lines YB-1 varies only slightly and amounts to about 20–30 ng per 15 µg of the total protein. The amount of *YB-1* mRNA shows no considerable variability either, ranging from 0.4 to 0.6 ng per 10 µg of the total RNA (Fig. 1C). The only exception is amounts of *YB-1* mRNA and YB-1 detected in 3T3 cells, where *YB-1* mRNA exceeds the level typical of other cell lines, while the amount of YB-1 does not approach it.

As to YB-1 and *YB-1* mRNA amounts in rabbit organ lysates, they differ from organ to organ (Fig. 1B). Rabbit testis, spleen, lungs, and heart are rich in YB-1 (30–40 ng per 50µg of the total protein), while liver, kidneys, brain, and muscles contain from 5 to 10 ng per 50µg of the total protein. The largest amount of *YB-1* mRNA was detected in testis (1.5 ng per 15 µg of the total RNA) and heart (0.5 ng per 15 µg of the total RNA). In other rabbit organs it varies from 0.1 to 0.2 ng per 15 µg of the total RNA. It should be noted that in spite of a low *YB-1* mRNA content in rabbit lungs and spleen, the YB-1 amount in these organs was almost as large as in testis, which suggested a high level of *YB-1* mRNA translation there or a high YB-1 stability.

### Analysis of *YB-1* mRNA Distribution between Polysomal and Free mRNP Fractions in Various Cell Lines

To analyze the translational level of *YB-1* mRNA, we determined its content in polysomal- and free mRNP fractions from lysates of various eukaryotic cell lines and rabbit organs. As a rule, the presence of mRNA in a polysomal fraction points to its efficient translation. In contrast, predominant localization of mRNA in a free mRNP fraction is indicative of the absence or low level of translation of this mRNA. The lysate samples were centrifuged through 50% sucrose to give free mRNPs in the supernatant, and polysomal mRNPs in the pellet. Isolation of the total RNA from these fractions was followed by detection of *YB-1* mRNA (or *GAPDH* mRNA as a control) by Northern blot. Applicability of this technique was verified by addition of up to 30 mM EDTA to the lysate prior to centrifugation (see Figure S1).

**Figure 2:**
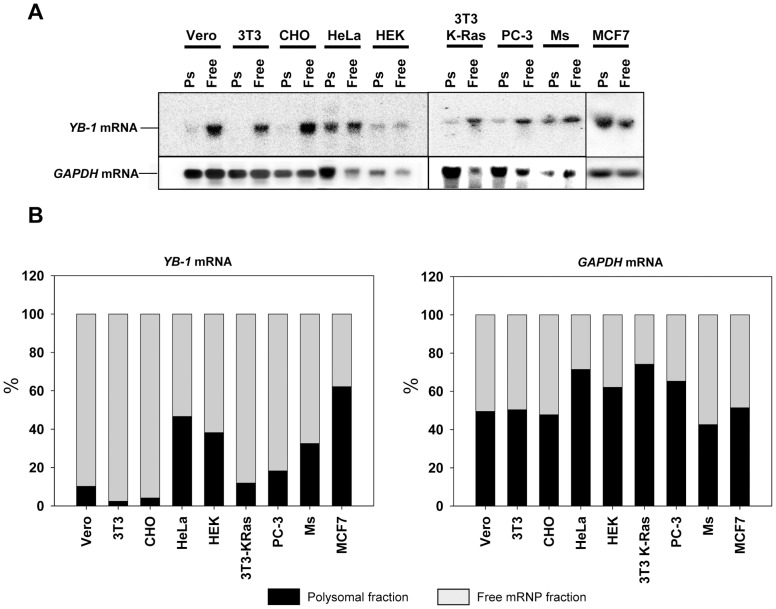
Analysis of *YB-1* mRNA distribution between polysomal and free mRNP fractions in various cell lines. A, Cells were scraped and lysed. Nuclei and mitochondria were removed by centrifugation, and cytosolic extracts were then spun through a 50% sucrose cushion at 100,000 rpm in a TLA-100 centrifuge (Beckman) for 13 min to separate postpolysomal supernatant from polysomes. Total RNA from postpolysomal supernatant and polysomal fractions (resuspended pellets) were extracted with TRIzol, subjected to agarose gel electrophoresis and Northern blot hybridization to [^32^P]-labeled *YB-1* and *GAPDH* cDNA. **B,** Relative radioactivity of the bands was determined using a Packard Cyclone Storage Phosphor System (Packard Instrument Company, Inc.). The sum of relative radioactivity values in free and polysomal mRNP fractions was taken to be 100%.

It follows from Fig. 2 that, predictably, in all these cell lines, the bulk (50–70%) of *GAPDH* mRNA was detected in translated polysomal mRNPs. A high content of *YB-1* mRNA in the polysomal fraction is typical of HeLa, MCF-7, and HEK293 cells, where about 50% of *YB-1* mRNA is localized to polysomal mRNPs. Other studied cell lines, including cancer cells, demonstrated predominant localization of *YB-1* mRNA in free mRNPs, which suggested its poor translation or no translation at all. In some samples (3T3 cells) more than 90% of *YB-1* mRNA is sedimented in the form of free mRNPs. Thus, in the majority of the studied cell lines, *YB-1* mRNA proved to be a weak template.

**Figure 3:**
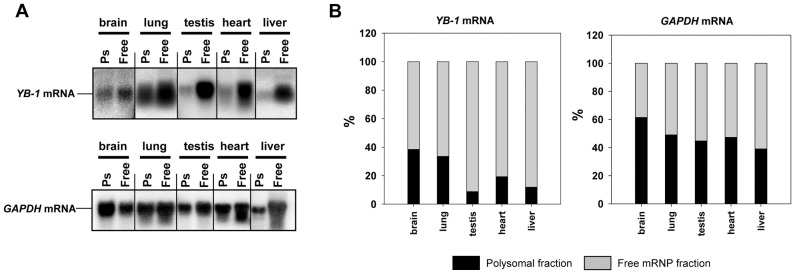
Analysis of *YB-1* mRNA distribution between polysomal and free mRNP fractions in rabbit organs. A, Tissue cytosolic extracts were spun through a 50% sucrose cushion at 100,000 rpm in a TLA-100 centrifuge (Beckman) for 13 min to separate postpolysomal supernatant from polysomes. Total RNA from postpolysomal supernatant and polysomal fractions (resuspended pellets) were extracted with TRIzol, subjected to agarose gel electrophoresis and Northern blot hybridization to [^32^P]-labeled *YB-1* and *GAPDH* cDNA. **B,** Relative radioactivity of the bands was determined using a Packard Cyclone Storage Phosphor System (Packard Instrument Company, Inc.). The sum of relative radioactivity values in free and polysomal mRNP fractions was taken to be 100%.

An analysis of *YB-1* mRNA distribution in lysates of rabbit organs showed a lower translational level of *YB-1* mRNA as compared to *GAPDH* mRNA used as a control (Fig. 3). However, there are some peculiarities worthy of note. Although the amount of *YB-1* mRNA in rabbit lungs is rather low, the level of its translation is presumably high enough to provide an amount of YB-1 exceeding that in other organs. In the brain, the translational level of *YB-1* mRNA is also high, but the amount of YB-1 is fairly low. This may be explained by enhanced YB-1 degradation possibly occurring in this organ. Testis contains large amounts of both *YB-1* mRNA and YB-1, with 90% of the former localized to free untranslated mRNPs. It can be expected that *YB-1* mRNA is stored as masked mRNPs.

### Assessment of YB-1 Synthesis in the Cell

**Figure 4:**
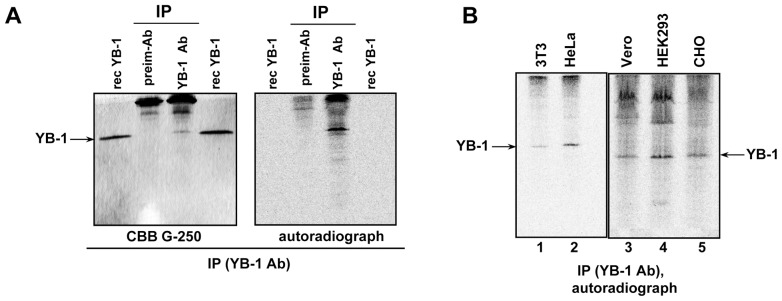
Assessment of YB-1 synthesis in the cell. A . HeLa cells were labeled with [^35^S]-methionine for 2 h, harvested and lysed. Cell lysate was used for immunoprecipitation with preimmune antibody or YB-1 antibody. Proteins bound to antibodies were resolved by acid-urea PAGE, stained with CBB G250, and the [^35^S]-labeled proteins were detected by autoradiography. Protein with an electrophoretic mobility corresponding to the recombinant YB-1 was cut out from the gel and identified by mass-spectrometry as YB-1. **B,** Assessment of YB-1 synthesis in cells of various lines. Cells were labeled using [^35^S]-methionine, harvested and lysed. Cell lysates were counterbalanced by radioactivity and used for immunoprecipitation with anti-YB-1 antibody. Proteins bound to antibodies were resolved by acid-urea PAGE, and the [^35^S]-labeled proteins were detected by autoradiography.

To confirm conclusions drawn from the experiments on cell cultures, we studied the rate of YB-1 synthesis in the cell. For this purpose, we proposed a technique implying metabolic cell labeling with [^35^S]-methionine followed by YB-1 immunoprecipitation with anti-YB-1 antibody, electrophoretic analysis of the precipitate, and autoradiography. Since YB-1 electrophoretic mobility in SDS gel electrophoresis shows no difference from that of heavy immunoglobulin chains, we used acid-urea electrophoresis. In these conditions, a much higher positive charge of YB-1 makes it show a higher electrophoretic mobility, as compared to the majority of co-precipitating proteins (including immunoglobulins). Figure 4A shows a typical electrophoregram and an autoradiograph obtained from these experiments. A protein with mobility corresponding to that of recombinant YB-1 was cut out from the gel, subjected to peptide fingerprinting, and identified as YB-1 (see Figure S2). Thus, the developed technique is applicable to assess YB-1 synthesis in eukaryotic cell cultures.

Using this technique, we analyzed YB-1 synthesis in the following cell lines: HeLa, HEK293 (with a large proportion of *YB-1* mRNA in the polysomal fraction), 3T3, CHO, and Vero (with a large proportion of *YB-1* mRNA in free mRNPs). The studied cell lysates were equilibrated in terms of radioactive labeling (incorporated [^35^S]-methionine). It appeared that in 3T3, CHO and Vero cells with a high level of *YB-1* mRNA in the free mRNP fraction YB-1 was nevertheless synthesized (Fig. 4B, lanes 1, 3 and 5), though to a lower extent than in HeLa and HEK293 cells (by 3 and 2 times, respectively) that have a high amount of *YB-1* mRNA in their polysomal fraction (Fig. 4B, lanes 2 and 4). So, the proportion of YB-1 synthesis in the total protein synthesis is lower for 3T3, Vero and CHO cells than for HeLa and HEK293 ones, which is in agreement with the above data on *YB-1* mRNA content in polysomal- and free mRNP fractions. Also, it can be stated that in the majority of the studied cell lines *YB-1* mRNA is poorly translated indeed, but not stored as untranslated masked mRNPs.

The proposed technique for detection of YB-1 synthesis in living cells is easy and informative as to some peculiar aspects of regulation of YB-1 amount in the cell, which have been yet poorly and only indirectly studied. Specifically, it allowed us to elucidate the relationship between YB-1 synthesis and the rate of cell division.

### YB-1 Synthesis and the Cell Division Rate

As known, the amount of YB-1 in a cell depends on its proliferative ability. It was found that an elevated YB-1 amount correlates with expression of proliferation markers [33], YB-1 overexpression causes hyperplasia [34], and cell doubling time increases with decreasing YB-1 expression under the action of siRNAs [35]. But the opposite may be speculated too, i.e., that YB-1 synthesis decreases as a result of a slower cell division.

A simple way to slow down cell division is to change cell density (or confluence) which underlies the so-called density-dependent inhibition of cell proliferation. We sought to see how an increased cell density (and hence, inhibition of cell proliferation) affected YB-1 synthesis. For this purpose 3T3 cells were cultivated up to a confluence of 40–50%, 80–90% and 100%, and additionally incubated with [^35^S]-methionine for 1 h, which provided label incorporation in direct proportion to the number of cells with appropriate density (see Figure S3).

**Figure 5:**
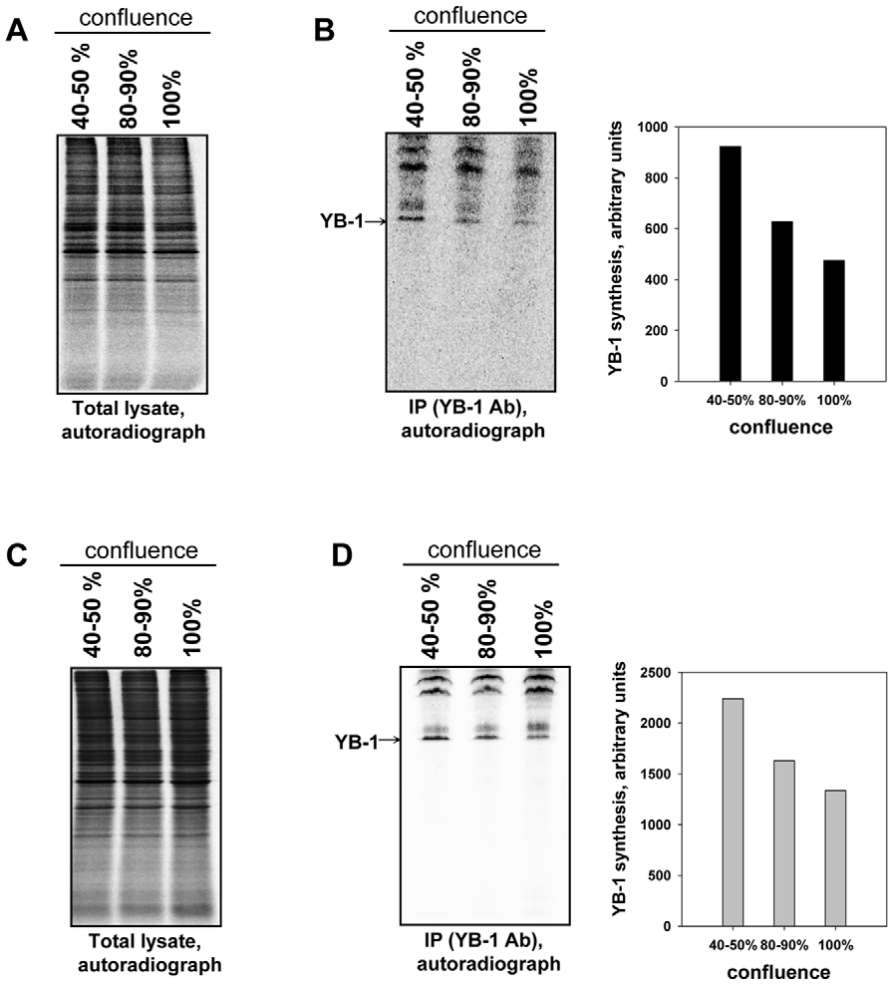
Dependence of YB-1 synthesis on cell confluence. NIH3T3 and HEK293 cells of various confluence were [^35^S]-methionine-labeled for 1 h, harvested and lysed. Cell lysates were counterbalanced by radioactivity (A and C) and used for immunoprecipitation with anti-YB-1 antibody. Proteins bound to antibodies were resolved by acid-urea PAGE, and [^35^S]-labeled proteins were detected by autoradiography (B and D). Relative radioactivity of the bands was determined using a Packard Cyclone Storage Phosphor System (Packard Instrument Company, Inc.).

Cell lysates from different dishes were equilibrated in terms of incorporated radioactive label ([^35^S]-methionine), and then YB-1 synthesis was analyzed (Fig. 5A). As appeared, an increase of confluence of 3T3 cells from 40% to 100% resulted in about 2-fold decrease of YB-1 synthesis (Fig. 5B). A similar result was shown by HEK293 cells (Figs. 5C and 5D).

**Figure 6:**
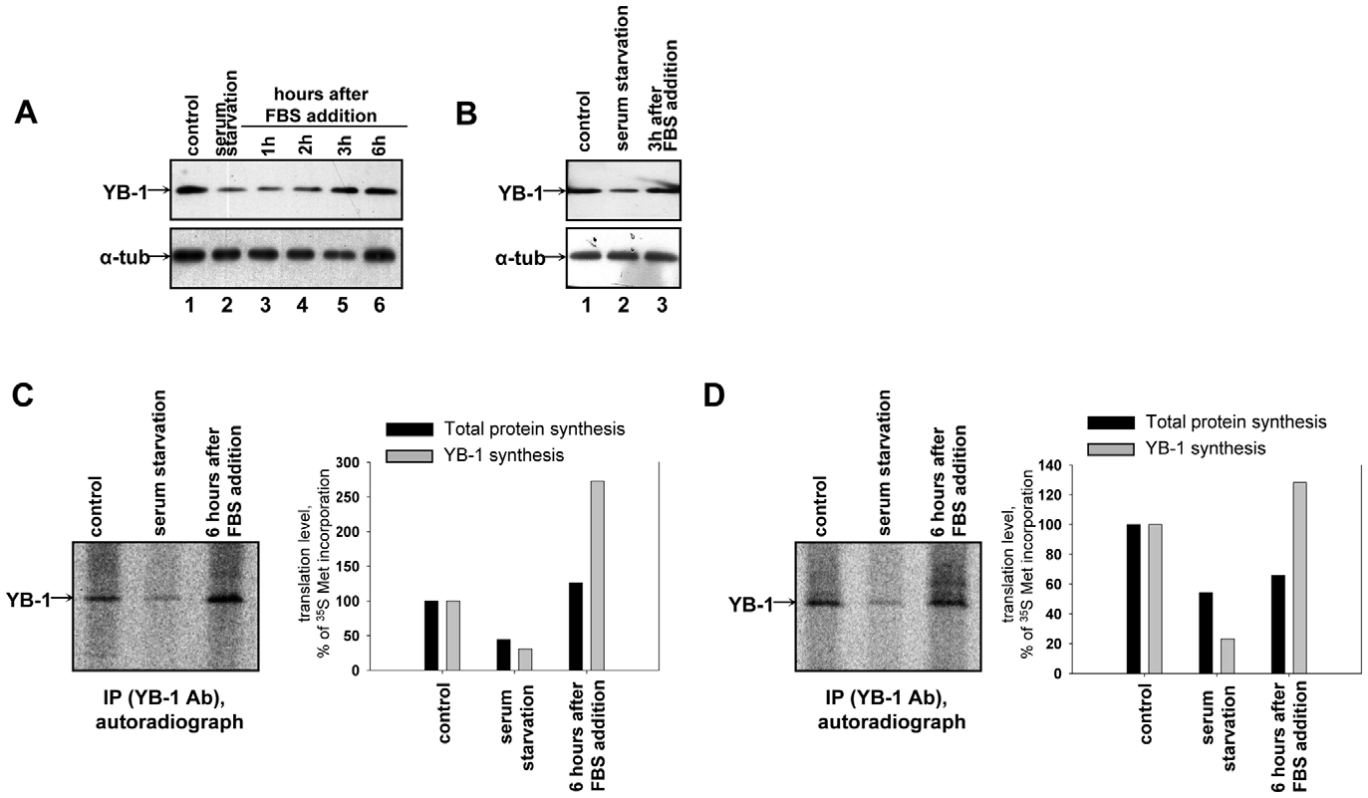
Renewal of YB-1 amount and YB-1 synthesis after serum starvation release. 3T3 (**A**) and HEK293(**B**) cells were serum starved (2 days). Cells were harvested at indicated time intervals after serum addition, lysed and used for Western blot analysis. 3T3(**C**) and HEK293(**D**) cells were serum starved (2 days). Control cells, serum starved cells and serum stimulated (6 h) cells were labeled with [^35^S]-methionine for 2 h, harvested and lysed. Cell lysates were used for immunoprecipitation with anti-YB-1 antibody. Proteins bound to antibodies were resolved by acid-urea PAGE, and [^35^S]-labeled proteins were detected by autoradiography. Relative radioactivity of the bands was determined using a Packard Cyclone Storage Phosphor System (Packard Instrument Company, Inc.). The level of YB-1 synthesis in cells without serum starvation was taken to be 100%.

Our other task was to reveal the effect of serum starvation (deficiency of growth factors) on YB-1 synthesis and its amount in the cell. After 2-day starvation 3T3 cells (Fig. 6A) and HEK293 cells (Fig. 6B) showed a decreased amount of YB-1 (cf. lanes 1 and 2) and a decrease in YB-1 synthesis that was more pronounced (4 or 5-fold) (Figs. 6C and 6D, cf. lanes 1 and 2) than the decrease of the total protein synthesis (about 2-fold) (Figs. 6C and 6D, bar graph). After serum addition to the starving 3T3 or HEK293 cells, the amount of YB-1 restored fairly soon (after 3–6 h) (Fig. 6A, lanes 3–6; Fig. 6B, lane 3). During this time YB-1 synthesis increased by 5 or 6 times and reached a level beyond that of control cells (Figs. 6C and 6D, cf. lanes 2 and 3), while the total protein synthesis was restored to the initial level in 3T3 cells and increased only slightly in HEK293 cells (Figs. 6C and 6D, bar graph).

### YB-1 Synthesis and the mTOR Signaling Pathway

What could be the reason for such a sensitivity of YB-1 synthesis to growth factors? More precisely, what signaling pathway provides it? The mTOR signaling cascade seems to be a most suitable candidate (see Figure S4). Its key protein mTOR is a protein kinase that acts as a kind of sensor that responds to nutrients, growth factors, or stress. With ample resources and the absence of stress, mTOR is active and phosphorylates some substrates, such as S6 kinase and 4E-BP [36]. This results in a higher level of protein synthesis, as well as cell growth and division. Apart from the overall regulation of translation, the mTOR signaling pathway is involved in specific regulation of translation of a large set(s) of mRNAs. Primarily, these are 5′TOP mRNAs and those with structured 5′UTR sensitive to 4E-BP which are responsible for cell growth and proliferation (reviewed in [37]). A question arises as to whether *YB-1* mRNA falls into the set of mRNAs whose translation is strongly dependent on activation of the mTOR signaling pathway. To answer it, we incubated HeLa and HEK293 cells with PP242, a specific mTOR inhibitor, for 2–3 h (the mRNA amount was shown to remain unchanged during this time interval [38]) and looked for an effect produced by this treatment on YB-1 synthesis.

**Figure 7:**
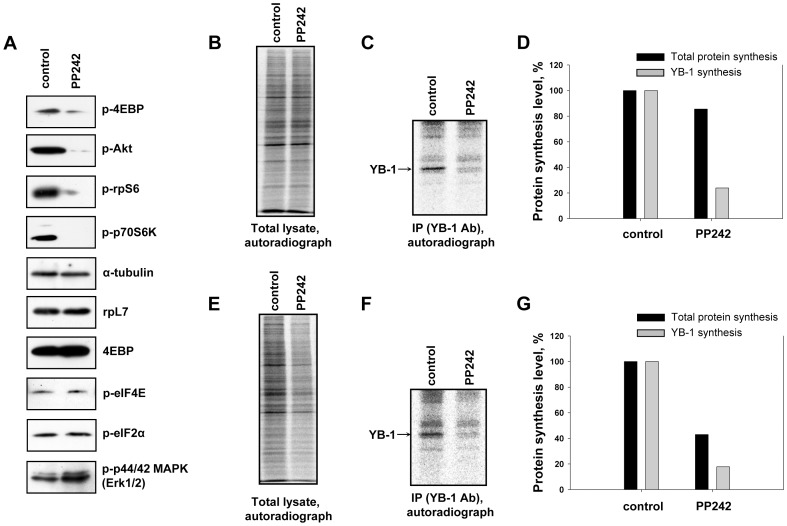
Effect of mTOR inhibitor PP242 on endogenous YB-1 synthesis in the cell. Untreated or PP242-treated (1 µM) HeLa (A, B, C, D) and HEK293 (E, F, G) cells were labeled with [^35^S]-methionine for 2 h, harvested and lysed. Cell lysates were counterbalanced by the total protein (B and E) and used for Western blotting (A) or immunoprecipitation with anti-YB-1 antibody. Proteins bound to antibodies were resolved by acid-urea PAGE, and the [^35^S]-labeled proteins were detected by autoradiography (C and F). Relative radioactivity of the bands was determined using a Packard Cyclone Storage Phosphor System (Packard Instrument Company, Inc.) (D and G). The level of YB-1 synthesis in cells without PP242 treatment was taken to be 100%.

Figure 7A shows that treatment of HeLa cells with PP242 (1µM) induced a significant decrease in phosphorylation of 4E-BP, p70S6 kinase (p70S6K), S6 ribosomal protein (rpS6) and Akt and had no effect on phosphorylation of eIF4E and eIF2α as it was previously reported [39]. Also, the total amount of 4E-BP remained unchanged.

At the same time PP242 had a slight effect on [^35^S]-methionine incorporation in the total protein of HeLa cells but made it 2 times lower in HEK293 cells (Figs. 7 B, E). However, in both HeLa (Figs. 7 C and D) and HEK293 cells (Figs. 7 F and G) the level of YB-1 synthesis was about 5 times lower.

**Figure 8:**
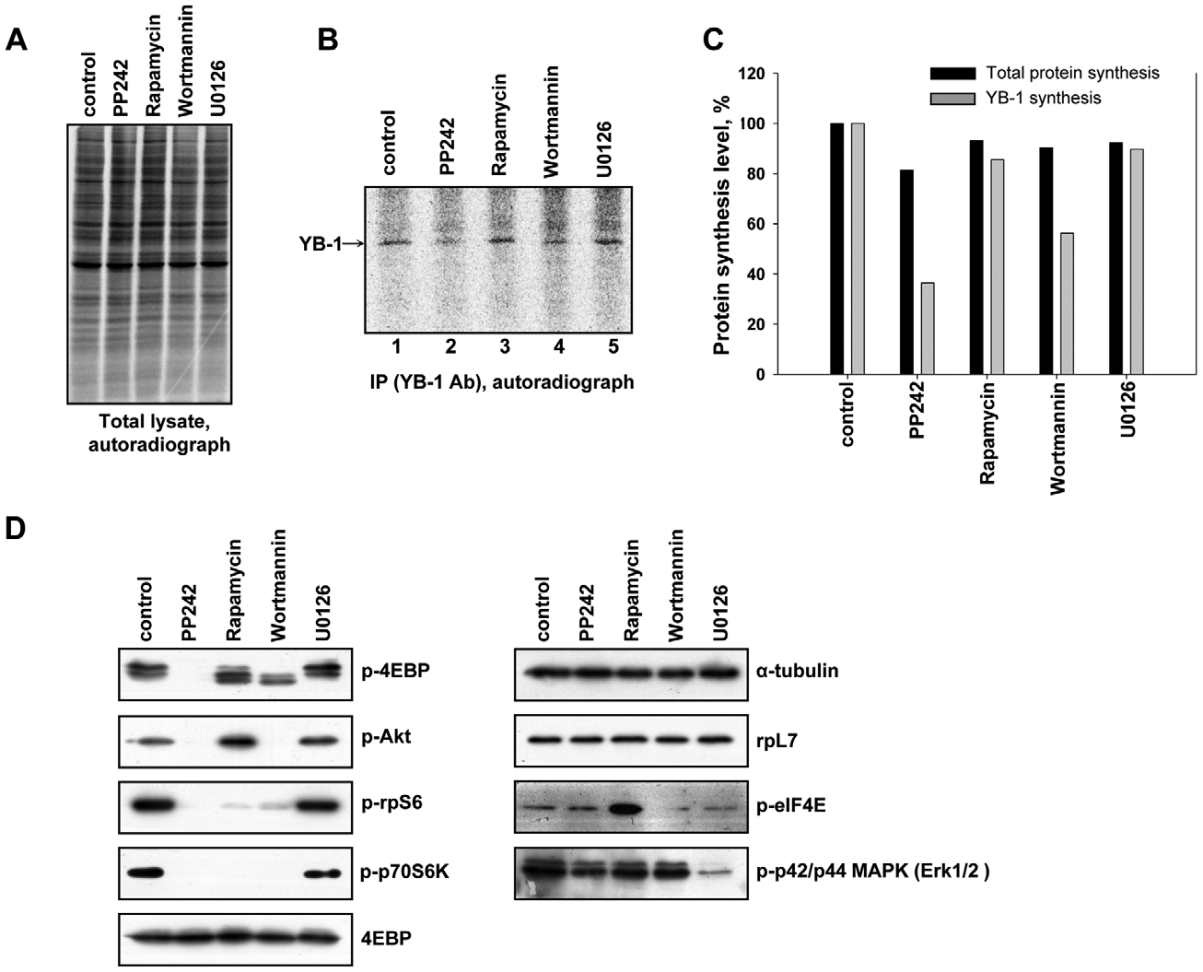
Effect of various cell signaling pathway inhibitors on endogenous YB-1 synthesis in the cell. Untreated (lane 1) or 1 µM PP242-treated (lane 2), or 0.1 µM rapamycin-treated (lane 3), or 0.5 µM wortmannin-treated (lane 4), or 10 µM U0126-treated (lane 5) HeLa cells were labeled with [^35^S]-methionine for 2 h, harvested and lysed. Cell lysates were counterbalanced by the total protein (**A**) and used for Western blotting (**D**) or immunoprecipitation with anti-YB-1 antibody. Proteins bound to antibodies were resolved by acid-urea PAGE, and [^35^S]-labeled proteins were detected by autoradiography (**B**). Relative radioactivity of the bands was determined using a Packard Cyclone Storage Phosphor System (Packard Instrument Company, Inc.) (**C**). The level of YB-1 synthesis in cells without drugs was taken to be 100%.

The use of other inhibitors of the mTOR signaling cascade gave the following results. Like PP242, wortmannin (an inhibitor of phosphoinositide 3-kinase (PI3K) suppressing phosphorylation of Akt and downstream substrates (Fig. 8D)) inhibited YB-1 synthesis (Figs. 8B (cf. lane 1 and lanes 2, 4) and 8C). U0126, inhibitor of Erk1/2 signaling pathway responsible, among other events, for eIF4E- and eIF4G phosphorylation (Fig. 8D) accompanied by translation stimulation [40], had no effect on YB-1 synthesis (Figs. 8B (cf. lanes 1 and 5) and 8C). What is of special interest, rapamycin, that affects the mTOR signaling pathway through reduced phosphorylation of S6 kinase but not 4E-BP (Fig. 8D), produced no effect on YB-1 synthesis either (Figs. 8B (cf. lanes 1 and 3) and 8C). As to the effect of the above inhibitors on total protein synthesis, it was insignificant (Figs. 8A and 8C). Together, these results show that regulation of YB-1 synthesis depends on the level of phosphorylated 4E-BP.

**Figure 9:**
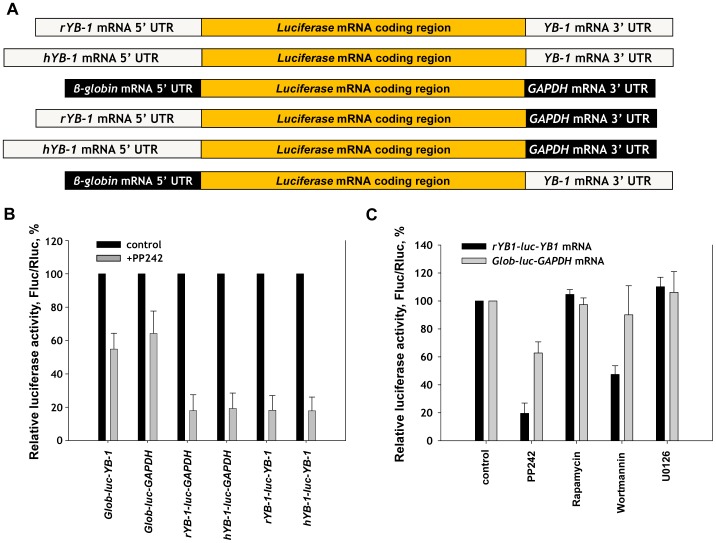
Effect of PP242 and other cell signaling pathway inhibitors on reporter mRNA translation in the cell. A. Scheme of mRNAs used here. *rYB-1* mRNA 5′ UTR denotes rabbit *YB-1* mRNA 5′ UTR (118 nt), *hYB-1* mRNA 5′ UTR denotes human *YB-1* mRNA 5′ UTR (171 nt), *GAPDH* mRNA 3′ UTR stands for *Glyceraldehyde 3-phosphate dehydrogenase* mRNA 3′ UTR. **B.** Untreated or PP242-treated (1 µM) HeLa cells were transfected by indicated reporter *Firefly luciferase* mRNA and *Renilla luciferase* mRNA (as internal control), cultivated for 2 h, harvested and assayed for *Firefly* and *Renilla* luciferase (*Fluc* and *Rluc*, respectively). **C.** Untreated or PP242-treated (1 µM), or rapamycin-treated (0.1 µM), or wortmannin-treated (0.5 µM), or U0126-treated (10 µM) HeLa cells were transfected by reporter *Firefly luciferase* mRNA (*rYB-1-luc-YB-1*) and *Renilla luciferase* mRNA (as internal control), cultivated for 2 h, harvested and assayed for *Firefly* and *Renilla* luciferase. The *Fluc*/*Rluc* ratio for the control (cells without treatment) was taken as 100%. Values are the means of at least three independent experiments. Errors are 2 standard deviations.

This dependence must be presumably underlain by 5′UTR (by analogy with 5′TOP and similar mRNAs). We sought to verify this suggestion using reporter mRNA coding for luciferase (luc) and possessing 5′UTR and/or 3′ UTR from *YB-1* mRNA. According to the NCBI database, *YB-1* mRNA 5′UTR may comprise from 118 to 171 nt. We used 2 variants of this 5′UTR: one of the minimal length – from rabbit mRNA (118 nt, (NM_001082785.1)) and the other of the maximal length – from human mRNA (171 nt, (NM_004559.3)). These or *luc* mRNA with control 5′- and 3′ UTRs (Fig. 9A) were used to perform transfection of HeLa cells pre-treated with pp242. After 2 h we estimated the level of translation of reporter mRNAs by enzymatic activity of luciferase. As seen from Fig. 9B, the mTOR inhibitor selectively inhibited translation of the reporter mRNAs containing rabbit- as well as human YB-1 mRNA 5′UTR but not luc mRNAs containing either the both control UTRs or solely *YB-1* mRNA 3′ UTR.

Besides, we checked the effect of other inhibitors on translation of the reporter mRNAs. As seen from Fig. 9C, similarly to PP242 and in contrast to other inhibitors used, wortmannin strongly and selectively inhibited translation of *luc* mRNAs with rabbit *YB-1* mRNA 5′ and 3′ UTRs. This result is perfectly consistent with results obtained for endogenous YB-1 synthesis (Fig. 8).

Thus, regulation of *YB-1* mRNA translation by the mTOR signaling pathway is determined by *YB-1* mRNA 5′ UTR. Its minimal possible length of about 118 nucleotides is sufficient for the function.

## Discussion

Here we studied the amounts of YB-1 and *YB-1* mRNA in eukaryotic cells of a number of cell lines and in various rabbit organs. In the cell lines these amounts vary only slightly. We have shown that in the majority of the studied cell lines *YB-1* mRNA is predominantly stored as free mRNPs, and hence, it is translated poorly, unlike the control *GAPDH* mRNA. This suggests that *YB-1* mRNA translation is sensitive to the amount or activity of some key cellular translation factor or a protein responsible for activity of this factor. Only some cells (cancer HeLa and MCF7 cells and embryonic HEK293 cells) provide conditions for a high level of *YB-1* mRNA translation.

On the other hand, analysis of the amounts of *YB-1* mRNA and YB-1 in rabbit organ lysates showed that they are variable and often display no correlation. In this situation, one can suggest several modes of post transcriptional regulation of YB-1 synthesis. Specifically, a high level of *YB-1* mRNA translation in the lungs explains a large amount of YB-1 along with a low content of *YB-1* mRNA in this organ. A high level of *YB-1* mRNA in the testis lysate might suggest a large amount of the protein. However, it is not so, suggesting that the bulk of *YB-1* mRNA may be stored in the masked form for a future use. In the brain, the level of *YB-1* mRNA translation is also high, but the YB-1 amount is fairly low. Possibly, this is underlain by an enhanced YB-1 degradation in this organ.

The developed here technique of YB-1 synthesis assessment allowed us to pioneer in finding the peculiarities of synthesis of endogenous YB-1 in the cell. Specifically, we report the dependence of YB-1 synthesis on the rate of cell division. As shown, the density-dependent inhibition of cell proliferation makes the rate of YB-1 synthesis much lower.

Serum starvation that also retards cell division results in a lower amount of YB-1 in the cell. As to YB-1 synthesis, it decreases to a larger extent than the total protein synthesis. The release from the starvation-induced arrest of cell division is accompanied by a rapid and essential increase of the YB-1 synthesis rate followed by renewal of the YB-1 amount in the cell.

The role of YB-1 in cell proliferation has been widely reported [33]–[35]. The main conclusion was that the proliferation rate depended on the YB-1 amount in the cell. Our experiments are evidence that, in turn, YB-1 synthesis depends on the proliferation rate. It should be stressed that endogenous YB-1 itself cannot trigger cell division. It is an intermediate on the signaling pathway(s) responsible for cell division, and its artificially altered amount can only shift the equilibrium required for signal transduction.

We have shown that the mTOR signaling pathway is one of the signaling cascades responsible for YB-1 synthesis regulation. Inhibition of the mTOR kinase by PP242 and the upstream kinase PI3K (by wortmannin) results in specific negative regulation of both endogenous YB-1 synthesis and translation of reporter constructs containing *YB-1* mRNA 5′ UTR. Besides, YB-1 insensitivity to rapamycin (mTOR inhibitor affecting mostly phosphorylation of S6 kinase and, to a lower extent, phosphorylation of 4E-BP) shows that it is phosphorylation of 4E-BP, and hence, its eIF4E-binding activity that regulates translation of YB-1. Of interest, the Erk1/2 inhibitor U0126 had no effect on YB-1 synthesis. It is known that ERK activation causes, in particular, phosphorylation of eIF4E by kinase Mnk1, and hence, an increase in translation of some eIF4E-dependent templates. The fact that YB-1 synthesis remained unaffected by ERK inhibition (and hence, inhibition of eIF4E phosphorylation) suggests that the level of *YB-1* mRNA translation depends on the eIF4E/phospho-4E-BP ratio rather than on the amount and phosphorylation of eIF4E.

The role of mTOR in regulation of translation of *YB-1* mRNA was supported by the results reported recently [38], [41]. Using ribosome profiling they showed that *YB-1* mRNA was detected in the pool of mRNAs whose translation was suppressed with inhibited mTOR. These authors believed that inhibition of *YB-1* mRNA was induced by PRTE (Pyrimidine Rich Translational Element) or TOP-like elements of *YB-1* mRNA 5′ UTR. But this element was positioned at the beginning of the 171 nt-long 5′ UTR of human *YB-1* mRNA, while our reporter construct contained a 5′UTR of rabbit mRNA which is shorter and lacks PRTE. However, *luc* mRNA with such a truncated 5′ UTR displayed sensitivity to inhibition of the mTOR signaling pathway. Presumably, the sequence responsible for sensitivity to mTOR inhibition within the 5′ UTR, is closer to the coding region than PRTE. It is still to be refined what elements of the primary or secondary structure of *YB-1* mRNA 5′UTR are responsible for mTOR-dependent regulation of *YB-1* mRNA translation.

## Materials and Methods

### Cell Cultures

3T3 and HEK293 cells (originally obtained from ATCC) were kindly provided by Dr Elena Nadezhdina (Institute of protein research RAS) and were cultivated in Dulbecco’s Modified Eagle’s Medium (DMEM) supplemented with 10% fetal calf serum, 2 mM glutamine, 100 U/ml penicillin, and 100 µg streptomycin (PanEco). HeLa cells were cultivated in DMEM/F12 supplemented with 10% fetal calf serum, 2 mM glutamine, 100 U/ml penicillin and 100 µg streptomycin (PanEco). The cells were incubated at 37°C in a humidified atmosphere containing 5% CO_2_ and passaged by standard methods.

In some experiments cells were treated with PP242 (Sigma-Aldrich, final concentration 1 µM), or rapamycin (Santa Cruz, final concentration 0.1 µM) or wortmannin (Santa Cruz, final concentration 0.5 µM) or U0126 (Santa Cruz, final concentration 10 µM) for 2–3 h.

### Immunoblotting

Samples for Western blot analysis were obtained as follows. The rabbit tissue samples were obtained from the Institute of Theoretical and Experimental Biophysics, Russian Academy of Sciences, where before use in experiment rabbits were kept under standard conditions of the barrier zone in compliance with Program of care and use of animals, and all manipulations with them were performed in compliance with ethical standards in animal research approved by the Institute’s Commission on Biological Safety and Ethics established on October 3, 2011 (# 173/k).

Rabbit tissues (brain, heart, spleen, lung, kidney, testis, liver and muscle) were homogenized using a Dounce homogenizer in SDS-electrophoresis sample buffer. The lysate was centrifuged at 5,000 *g* for 10 min to obtain cleared samples. The cultured cells were washed with phosphate buffered saline, lysed in SDS-electrophoresis sample buffer or buffer containing 20 mM HEPES-KOH (pH 7.6), 100 KCl, 5 mM MgCl_2_, 2 mM DTT, 0.25% Nonidet P-40, 0.2% SDS and protease inhibitors cocktail (Roche). The lysate was centrifuged at 5,000 *g* for 10 min to obtain cleared samples.

Proteins were separated by SDS-PAGE and transferred onto a nitrocellulose membrane. The membrane was blocked for 1 h at room temperature with non-fat 5% milk in TBS (10 mM Tris-HCl, pH 7.6, and 150 mM NaCl) and incubated overnight at 4°C in TBS-T (10 mM Tris-HCl, pH 7.6, and 150 mM NaCl, 0.05% Tween 20) supplemented with BSA (5%) and appropriate antibodies (polyclonal rat antibody against 14-aminoacid C-terminal peptide of YB-1 (IMTEK, Russia), polyclonal mouse antibody against α-tubulin (Sigma-Aldrich), polyclonal rabbit antibody against phospho-AKT^S473^ (Cell Signaling), phospho-p70S6K^T389^ (Cell Signaling), phospho-rpS6^S240/244^ (Cell Signaling), phospho-4EBP1^T37/46^ (Cell Signaling), 4EBP1 (Chemicon International Ink.). phospho-eIF4E^S209^ (Cell Signaling), phospho-eIF2α^S51^ (Cell Signaling), phospho-p44/42 MAPK (Erk1/2)^Thr202/Tyr204^ (Cell Signaling), rpL7 (Abcam)).

Immunocomplexes were detected using an ECL Prime kit (GE Healthcare) according to the manufacturer’s recommendations.

### Northern Blotting Analysis

Total RNA from cells or polysomal and post-polysomal fractions of lysates was extracted by the TRIzol method. Total RNA from rabbit tissues was extracted by homogenizing with TRIzol using a Dounce homogenizer. 10–15 µg of total RNA from cells or tissues (measured by A_260_) was separated by electrophoresis on a denaturing 1.5% agarose gel containing 2.2 M formaldehyde and 1x MOPS buffer (40 mM MOPS, 10 mM sodium acetate, 1 mM EDTA, pH 7.0). RNA was transferred onto a nylon membrane (Hybond-N, GE Healthcare) and crosslinked using a transilluminator-cross-linker (Vilber-Lourmat) at 0.15 J/cm^2^. Membrane-bound RNA was hybridized to a 1055 nt fragment of *YB-1* cDNA probe (nt 1–1055, GeneBank U16821.1) or a 466 nt fragment of *GAPDH* cDNA probe (nt 870–1336, NM_001256799.1) labeled with [^32^P]dATP (2,000 Ci/mM; IBCh, Russia) using a DecaLabel DNA labeling kit (Fermentas) in hybridization buffer (0.5 M KH_2_PO_4_/K_2_HPO_4_, pH 7.4, 7% SDS, 10 mM EDTA) at 65°C for 12–16 h. The membrane was washed twice with 2x SSC, 0.1% SDS for 5 min at room temperature, twice with 0.2x SSC, 0.1% SDS for 5 min at RT, twice with 0.2x SSC, 0.1% SDS for 15 min at 42°C, and twice with 0.1x SSC, 0.1% SDS for 15 min at 68°C, and analyzed by autoradiography. If needed, the relative radioactivity of the bands was determined using a Packard Cyclone Storage Phosphor System (Packard Instrument Company, Inc.).

### Analysis of mRNA Distribution between Polysomes and Free mRNPs

Cells were washed twice with ice-cold PBS containing 0.1 mg/ml cycloheximide and lysed directly on the plate after addition of 400 µl of polysome extraction buffer: 15 mM Tris-HCl, pH 7.4, 15 mM MgCl_2_, 0.3 M NaCl, 1% Triton X-100, 0.1 mg/ml cycloheximide and 1 mg/ml heparin, 0.2 mM VRC (vanadyl ribonucleoside complex). Rabbit tissues were homogenized using a Dounce homogenizer in the same buffer. Extracts were transferred into 1.5 ml tubes and incubated on ice for 10 min with occasional mixing. The nuclei and debris were removed by centrifugation at 12,000 *g* for 10 min in a microcentrifuge. Supernatants were recovered, and 200 µl aliquots were layered onto 50 µl of 50% sucrose cushion composed of extraction buffer lacking Triton X-100 and pelleted at 90,000 rpm for 13 min in a TLA-100 rotor (Beckman) at 4°C (13 min was enough to have all ribosomes pelleted, while all 40S ribosomal subunits were still retained in supernatant (see Figure S1)). RNA from supernatant (free mRNPs) and pellet (polysomal mRNPs) were isolated by TRIzol and analyzed by Northern blotting.

### Metabolic [^35^S]-labeling of Cell Proteins and Immunoprecipitation (IP)

For [^35^S]-methionine labeling, the cells were cultivated in DMEM lacking L-methionine but containing 0.1 mCi/ml of L-[^35^S]-methionine (Perkin Elmer, 1,000 Ci/mmol) for 1–2 h. The cells were washed with phosphate buffer saline, scraped and lysed with buffer containing 20 mM HEPES-KOH, pH 7.6, 100 KCl, 5 mM MgCl_2_, 2 mM DTT, 0.25% Nonidet P-40, 0.2% SDS and protease inhibitors cocktail. Cell debris was removed by centrifugation at 10,000 g for 15 min, and extracts were then directly utilized in IP experiments. For IP, cell extracts were incubated with appropriate antibodies (polyclonal rat antibody against 14-aminoacid C-terminal peptide of YB-1 (IMTEK, Russia) or rat pre-immune antibodies, 100 µg each) immobilized on protein G-Sepharose beads (GE Healthcare) for 2 h at 4°C. After extensive washing with PBS, proteins were eluted with acid-urea sample buffer (8 M urea, 5% acetic acid, 0.025 methylene blue), and analyzed by acid-urea 10% polyacrylamide gel electrophoresis and autoradiography. The relative amount of radioactivity in the bands was determined using a Packard Cyclone Storage Phosphor System (Packard Instrument Company, Inc.).

### Plasmid Construction

Plasmids pSP36T-5′UTR*YB1*-*Fluc*-3′UTR*YB1*A50 and pSP36T-5′UTR *b-globin-FLuc*-3′UTR*GAPDH*A50 for synthesis of reporter mRNAs were obtained on the basis of pSP36T*Luc*A(50) [42] as described in Protocol S1).

### *In vitro* Transcription

Firefly *luciferase* (*Fluc*) cap^−^poly(A)^+^ mRNA with 5′ and 3′ UTRs from *YB-1* mRNA was transcribed by SP6 RNA polymerase from pSP36T-5′UTR*YB1*-*Fluc*-3′UTR*YB1*A50 linearized with *HpaI*.

Firefly *luciferase* cap^−^poly(A)^+^ mRNA with 5′ UTR from *beta-globin* mRNA and 3′ UTR from *GAPDH* mRNA was transcribed by SP6 RNA polymerase from pSP36T-5′UTR *b-globin-Fluc*-3′UTR*GAPDH*A50 linearized with *HpaI*.

*Renilla luciferase* (*Rluc*) mRNA was obtained as described by [43].

The transcription was performed as described previously [44].

Capped mRNA transcripts were obtained using a ScriptCap™ m^7^G Capping System and ScriptCap 2′-O-Methyltransferase Enzyme (CellScript) according to the manufacturers’ recommendations.

### Transient RNA Transfection of Cells and Luciferase Activity Detection

For transfection experiments, HeLa cells were placed onto a 24-well dish for 24 h prior to their transfection by reporter *luciferase* mRNAs. The transfection was performed using Lipofectamine 2000 (Invitrogen). For a typical RNA transfection, 0.5 µg of Fluc mRNA and 0.05 µg of *Rluc* mRNA were incubated with 2 µl of the transfection reagent in 100 µl DMEM for 20 min and then added to the growth medium. 1.5 h later, the cells were harvested, and luciferase activities were analyzed using a Dual Glo Luciferase Assay kit (Promega). All these transfections were repeated several times.

## Supporting Information

Figure S1 Analysis of mRNA distribution between polysomal and free mRNP fractions in the cell. HEK293 cells were scraped and lysed. Nuclei and mitochondria were removed by centrifugation, and cytosolic extracts without (lanes 1 and 2) or with (lanes 3 and 4) 30 mM EDTA were then spun through a 50% sucrose cushion at 100,000 rpm in a TLA-100 centrifuge (Beckman) for 13 min to pellet polysomes. Total RNA from postpolysomal supernatant and polysomal fractions (resuspended pellets) were extracted with TRIzol, subjected to agarose gel electrophoresis and Northern blot hybridization to [^32^P]-labeled *GAPDH* cDNA. Approximately 65% of *GAPDH* mRNA was detected in polysomal pellet. In the presence of 30 mM EDTA, the entire *GAPDH* mRNA was found in supernatant. Hence, in the absence of EDTA the entire *GAPDH* mRNA detected in the polysomal fraction was bound to mono- and polysomes. 18S rRNA was absent too from the polysomal fraction after EDTA treatment. This means that all particles under 40S were retained in supernatant.
(TIF)

Figure S2 Results of peptide mass fingerprint analysis of the protein cut out from the gel after anti-YB- 1 Ab IP and acid-urea PAGE.
(TIF)

Figure S3 Dependence of radioactive label ([^35^S]-Met) incorporation on cell confluence. NIH3T3 cells of various confluence were [^35^S]-methionine-labeled, harvested and lysed. Cell lysates were analyzed by PAGE and autoradiography Relative radioactivity of the bands was determined using a Packard Cyclone Storage Phosphor System (Packard Instrument Company, Inc.)
(TIF)

Figure S4 A simplified scheme of the mTOR signaling pathway. The basic mTOR substrates are 4E-BP (eukaryotic initiation factor 4E binding protein) and S6 kinase (p70S6K). Phosphorylation of 4E-BP results in its lower affinity for eIF4E, which makes the latter accessible for translation initiation. Activated S6 kinase phosphorylates a number of substrates (eukaryotic translation initiation factor 4B (eIF4B), ribosomal protein S6, programmed cell death 4 (PDCD4) - a tumor suppressor that binds to eIF4A, eukaryotic translation elongation factor 2 kinase (eEF2K) etc.), thereby contributing to activation of both initiation and elongation of translation. Inhibitors of mTOR kinase are rapamycin and PP242, the former predominantly inhibiting phosphorylation of p70S6K. The major mTOR activating pathway is the PI3K/Akt kinase cascade. Its inhibition with wortmannin affects, among others, mTOR kinase. Inhibition of Erk kinase with U0126 causes inhibition of Mnk-1, and hence, suppression of eIF4E phosphorylation, thereby decreasing translation of some eIF4E-sensitive mRNAs (here used as a control).
(TIF)

Protocol S1 Plasmid construction.
(DOCX)
