# Supplementary figures and images for: YB-1 Synthesis Is Regulated by mTOR Signaling Pathway
Source: PLoS One. 2012 Dec 20;7(12):e52527. doi: 10.1371/journal.pone.0052527 (PMC3527543; doi:10.1371/journal.pone.0052527)

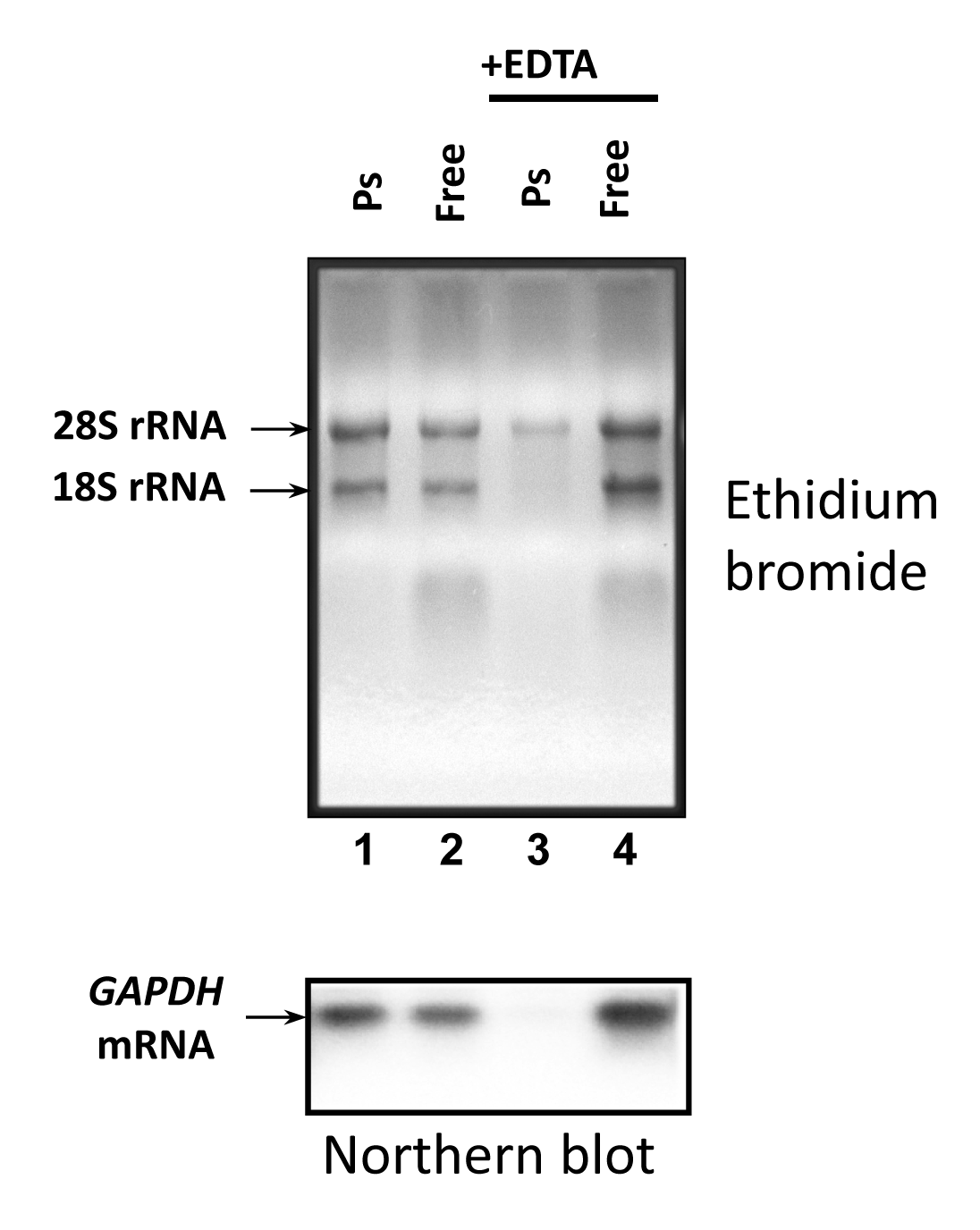

Supplement: Figure S1 — Analysis of mRNA distribution between polysomal and free mRNP fractions in the cell. HEK293 cells were scraped and lysed. Nuclei and mitochondria were removed by centrifugation, and cytosolic extracts without (lanes 1 and 2) or with (lanes 3 and 4) 30 mM EDTA were then spun through a 50% sucrose cushion at 100,000 rpm in a TLA-100 centrifuge (Beckman) for 13 min to pellet polysomes. Total RNA from postpolysomal supernatant and polysomal fractions (resuspended pellets) were extracted with TRIzol, subjected to agarose gel electrophoresis and Northern blot hybridization to [32P]-labeled GAPDH cDNA. Approximately 65% of GAPDH mRNA was detected in polysomal pellet. In the presence of 30 mM EDTA, the entire GAPDH mRNA was found in supernatant. Hence, in the absence of EDTA the entire GAPDH mRNA detected in the polysomal fraction was bound to mono- and polysomes. 18S rRNA was absent too from the polysomal fraction after EDTA treatment. This means that all particles under 40S were retained in supernatant. (TIF) [file pone.0052527.s001.tif]

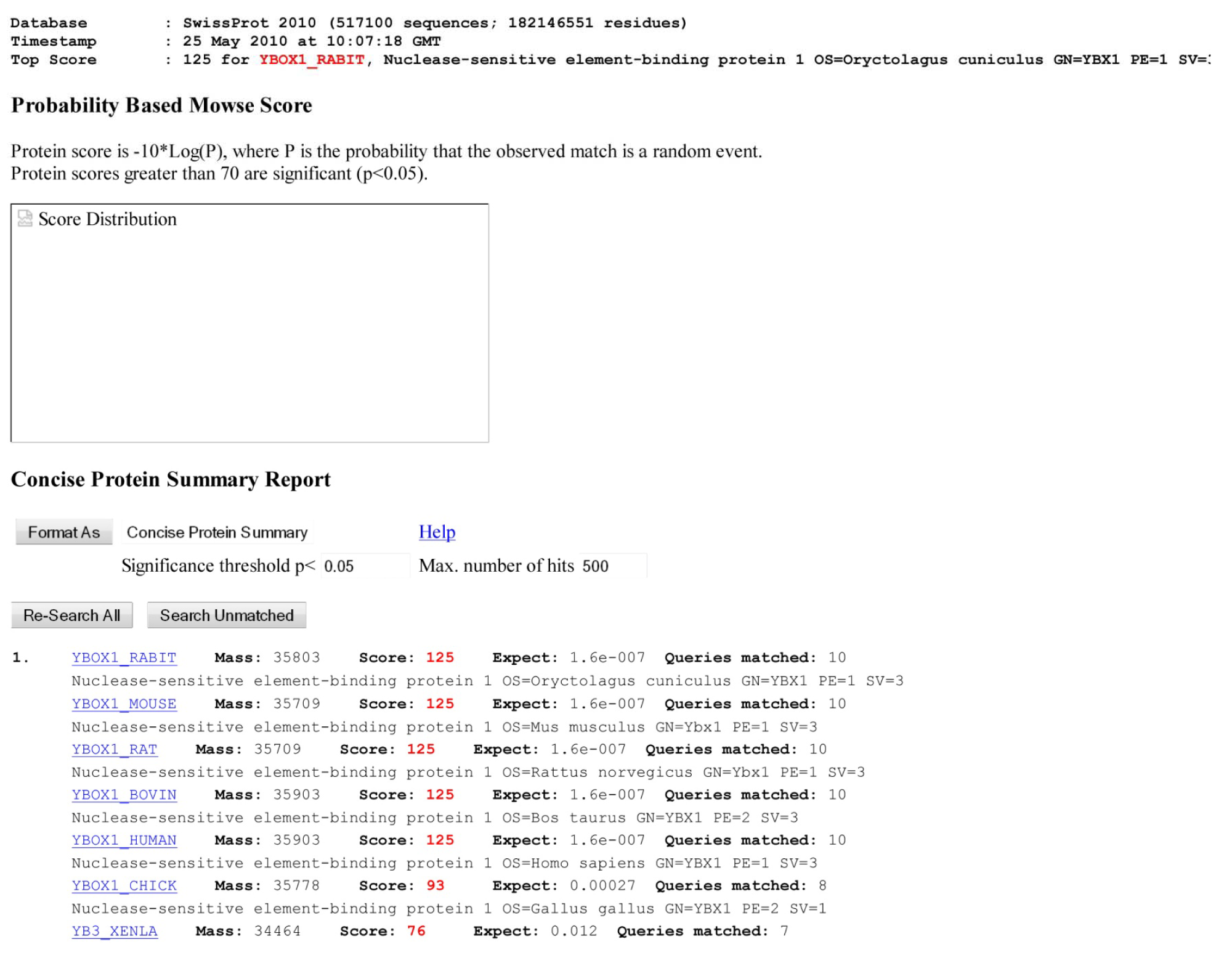

Supplement: Figure S2 — Results of peptide mass fingerprint analysis of the protein cut out from the gel after anti-YB- 1 Ab IP and acid-urea PAGE. (TIF) [file pone.0052527.s002.tif]

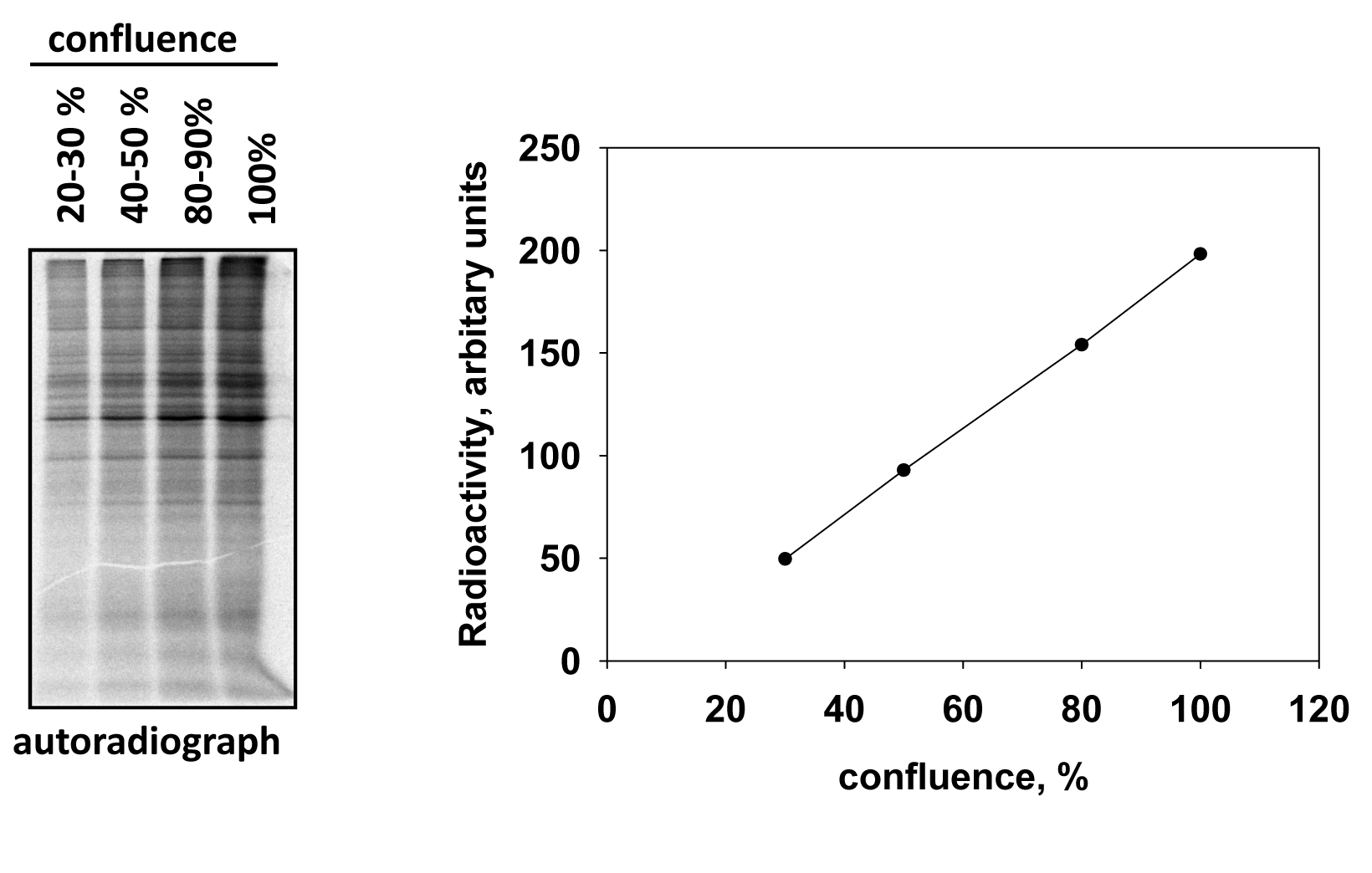

Supplement: Figure S3 — Dependence of radioactive label ([35S]-Met) incorporation on cell confluence. NIH3T3 cells of various confluence were [35S]-methionine-labeled, harvested and lysed. Cell lysates were analyzed by PAGE and autoradiography Relative radioactivity of the bands was determined using a Packard Cyclone Storage Phosphor System (Packard Instrument Company, Inc.) (TIF) [file pone.0052527.s003.tif]

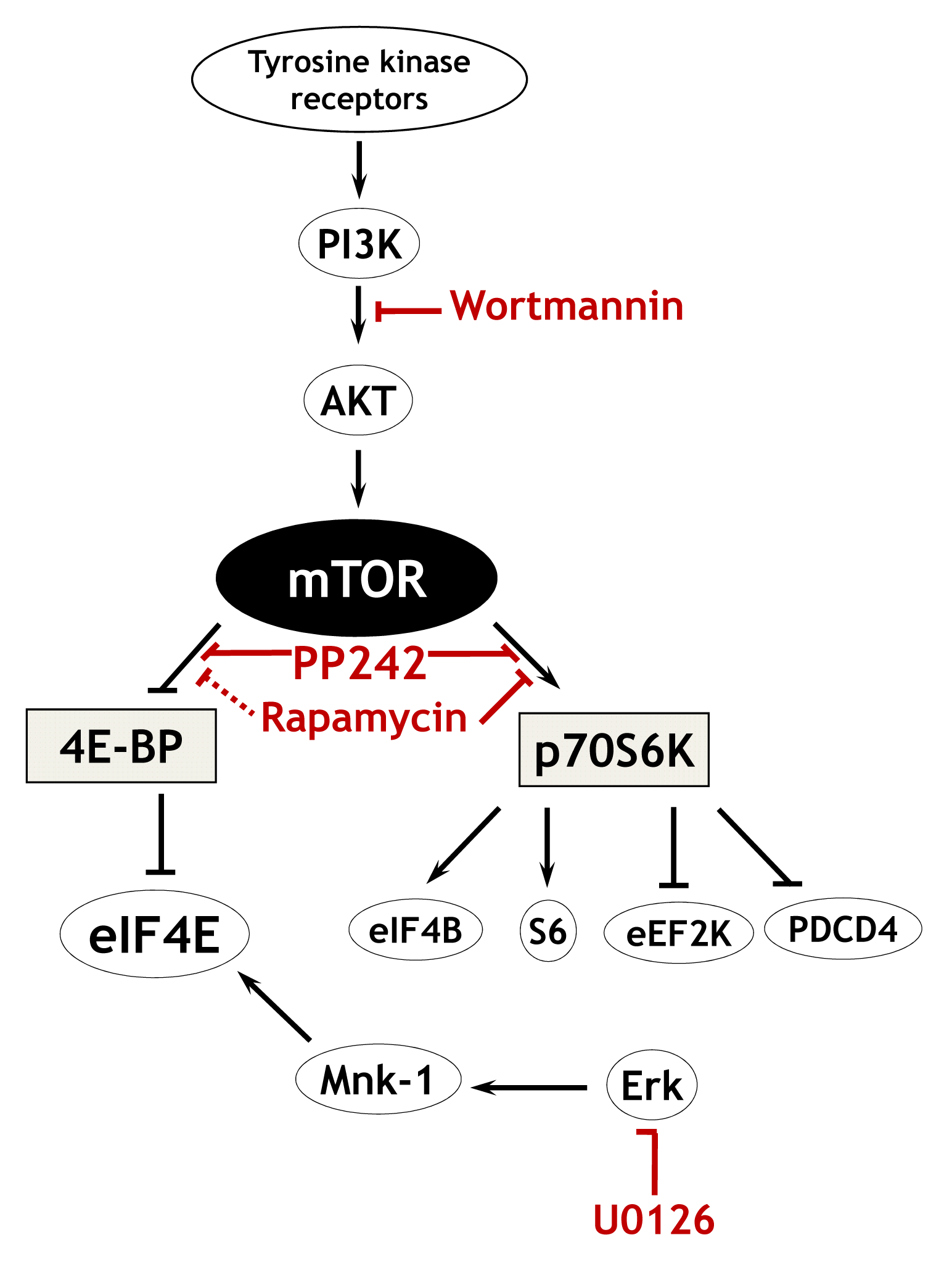

Supplement: Figure S4 — A simplified scheme of the mTOR signaling pathway. The basic mTOR substrates are 4E-BP (eukaryotic initiation factor 4E binding protein) and S6 kinase (p70S6K). Phosphorylation of 4E-BP results in its lower affinity for eIF4E, which makes the latter accessible for translation initiation. Activated S6 kinase phosphorylates a number of substrates (eukaryotic translation initiation factor 4B (eIF4B), ribosomal protein S6, programmed cell death 4 (PDCD4) - a tumor suppressor that binds to eIF4A, eukaryotic translation elongation factor 2 kinase (eEF2K) etc.), thereby contributing to activation of both initiation and elongation of translation. Inhibitors of mTOR kinase are rapamycin and PP242, the former predominantly inhibiting phosphorylation of p70S6K. The major mTOR activating pathway is the PI3K/Akt kinase cascade. Its inhibition with wortmannin affects, among others, mTOR kinase. Inhibition of Erk kinase with U0126 causes inhibition of Mnk-1, and hence, suppression of eIF4E phosphorylation, thereby decreasing translation of some eIF4E-sensitive mRNAs (here used as a control). (TIF) [file pone.0052527.s004.tif]
