# Supplementary material for: YB-1 Synthesis Is Regulated by mTOR Signaling Pathway
Source: PLoS One. 2012 Dec 20;7(12):e52527. doi: 10.1371/journal.pone.0052527 (PMC3527543; doi:10.1371/journal.pone.0052527)
Supplement: Protocol S1 — Plasmid construction. (DOCX) [file pone.0052527.s005.docx]

**Protocol S1.**

**Plasmid construction.**

Plasmids pSP36T-5’UTR *YB1-Fluc*-3’UTR *YB1* A50 and pSP36T-5’UTR *b-globin-Fluc*-3’UTR *GAPDH* A50 were obtained as follows.

The 3’ UTR of *YB-1* mRNA was obtained by PCR amplification of pBluescript II SK *YB-1* WT. The forward primer was 5’- AGCAGGGCGGGG**AGATCT**AAATGCCGGCTTAA -3’, the reverse primer was 5’‑ GGGCTGCAGGAATTC**GCGGCCGC**CTTTATTA -3’ (the *BglII* and *NotI* restriction sites are bold, the stop codon is underlined). The PCR product was ligated into the pBluescript II KS (Fermentas) treated with *EcoRV*. A clone with (+) orientation was used. The construct was named pBluescript II KS #1.

The *Fluc* cDNA was obtained by PCR amplification of pSP36T-5’UTR *b-globin-Fluc*A50 (Wakiyama et al., 1997). The forward primer was 5’‑GTCCATATGGAAGACGCCAAAAACATAAAGAAA‑3’, the reverse primer was 5’‑CATTTT**AGATCT**TGGACTTTCCGCCCTT‑3’ (the *BglII* restriction sites are bold, the stop codon is underlined). The PCR product was ligated into the pBluescript II KS (Fermentas) treated with *EcoRV*. A clone with (+) orientation was used. The construct was named pBluescript II KS #2.

The 3’ UTR of *YB-1* mRNA was cut off pBluescript II KS #1 by digesting with *BglII* and *NotI* (i.e., it was treated with T4 DNA Polymerase for blunting) and ligated into pBluescript II KS #2 treated with *BglII* and *SalI* (subjected to T4 DNA polymerase-induced blunting). The resultant construct was named pBluescript II KS #3.

The Fluc with 3’ UTR from *YB-1* mRNA was cut off pBluescript II KS #3 by digesting with *XbaI* and *KpnI* (blunted by T4 DNA polymerase) and ligated into pSP36T-5’UTR *b-globin-Fluc* A50 treated with *XbaI* and *BglII* (blunted by T4 DNA polymerase). The resultant construct was named pSP36T #4.

Substitution of the restriction site *HpaI* for *Sma I* (after polyA tail) was obtained by PCR amplification of pSP36T #4. The forward primer was 5’‑GTCCATATGGAAGACGCCAAAAACATAAAGAAA‑3’, the reverse primer was 5’‑CCGACCTGCAG**GTTAAC(**T_50_)AGATCCG‑3’ (the *HpaI* restriction sites are bold, the *PstI* restriction site is underlined). The PCR product was treated with *XbaI* and *PstI* and ligated into pSP36T #4 treated with the same restriction endonucleases. The resultant construct was named pSP36T #5.

The 3’ UTR of *GAPDH* mRNA was obtained by PCR amplification of cDNA of HeLa cells. The forward primer was 5’‑ATGGCCTCC**AGATCT**AAGACCCCTG‑3’, the reverse primer was 5’‑CTCGAG**CTCGAG**GGTTGAGCACAGGGT‑3’ (the *BglII* and *XhoI* restriction sites are bold, the stop codon is underlined). The PCR product was treated with *BglII* and *XhoI* and ligated into pSP36T #5 treated with the same restriction endonucleases. The resultant construct was named pSP36T-5’UTR *b-globin-Fluc*-3’UTR *GAPDH* A50.

The 5’ UTR from rabbit *YB-1* mRNA was obtained by PCR amplification of pBluescript II SK *YB-1* WT. The forward primer was 5’‑TAATACGACTCACTATAGGG‑3’, the reverse primer was 5’‑CTGC**CCATGG**TTGCGGTGATGGTGACTG‑3’ (the *NcoI* restriction site is bold, the start codon is underlined). The 5’ UTR from human *YB-1* mRNA was obtained by PCR amplification of total cDNA from HeLa cells. The forward primer was 5’‑GTCTCGAGGGCTTATCCCGCCTGTC‑3’, the reverse primer was 5’‑CTGC**CCATGG**TTGCGGTGATGGTGACTG‑3’ (the *NcoI* restriction site is bold, the start codon is underlined). The PCR products was treated with *HindIII* and *NcoI* and ligated into pSP36T-5’UTR *b-globin-Fluc*-3’UTR *GAPDH* A50 or pSP36T #5 treated with the same restriction endonucleases. The resultant constructs was named pSP36T-5’UTR r*YB1-Fluc-*3’UTR *GAPDH* A50, pSP36T-5’UTR h*YB1-Fluc-*3’UTR *GAPDH* A50, pSP36T-5’UTR r*YB1-Fluc-*3’UTR *YB1* A50 and pSP36T-5’UTR h*YB1-Fluc-*3’UTR *YB1* A50.
